# Face-to-face and online teaching experience on experimental animals and alternative methods with nursing students: a research study

**DOI:** 10.1186/s12912-023-01172-5

**Published:** 2023-01-13

**Authors:** Juan F. Garcia Sierra, M. Nélida Fernandez Martinez, Cristina Lopez Cadenas, Raquel Diez Laiz, José M. Rodriguez Lago, Ana M. Sahagun Prieto

**Affiliations:** 1 Computer Architecture and Technology, Department of Mechanical, Informatics and Aerospatiale Engineering, School of Industrial and Computer Engineering. University of Leon, Leon, Spain; 2 Pharmacology, Department of Biomedical Sciences, Institute of Biomedicine (IBIOMED). Faculty of Health Sciences, University of Leon, Leon, Spain

**Keywords:** Alternative method, Experimental animals, Interdisciplinary collaboration, Nursing studies, Pharmacology

## Abstract

**Background:**

Animal models are increasingly used in Nursing science to study care approaches. Despite the scientific relevance and the ethical debate surrounding the use of experimental animals, there is a scarcity of scholarly literature exploring this topic in Nursing Schools.

**Aim:**

To evaluate perceptions and attitudes of nursing students enrolled in a Pharmacology course on the use of experimental animals and implementation of alternative methods, by comparing the experience for two academic years. An interdisciplinary collaboration has also been developed.

**Methods:**

A descriptive cross-sectional, quantitative study was developed. Undergraduate nursing students were enrolled in the Pharmacology subject at the University of Leon (Spain). The study was carried out in the Pharmacology facilities. Students followed a two-session practical class regarding experimental animals and alternative methods in the Pharmacology course (Degree in Nursing) in two different academic years (2019–20/2020–21). At the end of the activity, they answered a questionnaire to assess their opinions on the use of experimental animals and alternative methods in Pharmacology and the 3Rs principle.

**Results:**

A comparison of the students’ perception with and without direct participation in the evaluation of promazine effects in mice was made. A total of 190 students participated in the teaching experience, providing high scores in all items (4–5 out of 5 points) regarding the teaching experience. Students became also aware of the advantages and disadvantages on the use of experimental animals, as well as the ethical considerations to bear in mind for their use and the need for alternative methods.

**Conclusions:**

In the students’ opinion, the total replacement of animals by alternative techniques was very difficult, and they preferred to do the practice face-to-face. The alternative method designed was useful for the students to accept the employment of experimental animals in biomedical research and education, and know the legislation applied in the protection of animals.

## Background

Experimental animals are used for a wide range of scientific research (e.g., new drugs and vaccines development, safety assessment of chemical products) and for education [1, 2]. They are necessary to meet the needs of society regarding health problems, food control and environmental toxicity.

The use of experimental animals has been the subject of very complex ethical debates for a long time, leading to legal regulations for its protection in different countries. Directive 2010/63/EU [3] regulates their use in research and teaching in the European Union countries and, in the introduction section, it is indicated that “*while it is desirable to replace the use of live animals in procedures by other methods not entailing the use of live animals, the use of live animals continues to be necessary to protect human and animal health and the environment*”. Thus, experimental animal testing is required prior to the approval of new drugs and vaccines.

Although initially nursing knowledge does not seem to be related to animal experimentation, nursing research covers a wide range of topics related to human health, and experimental animals can provide a wealth of information that may then be applied to human beings. Nurses may provide a discipline-specific focus on animal studies, and improve the pertinency of these studies for nursing practice [4]. Thus, it is necessary to raise awareness and promote the application of animal experimentation in various fields of nursing research, as a way to expand the body of nursing knowledge applicable to the same conditions in humans, and to develop nursing interventions [5].

In this sense, a small but increasing number of nurse researchers have used animals for decades to study diseases [6], and to find solutions for patient care questions. Nevertheless, its use in nursing research is still controversial, as it is under debate whether all studies carried out by nurses (including those developed with animal models) should be based on nursing conceptual frameworks, and the extent to which this type of research may contribute to nursing knowledge [7–9]. As Page [9] noted, the nursing discipline should embrace animal research as an integral component of its own research.

The use of experimental animals in the University education is small compared to those employed for research purposes, and it has decreased considerably in recent years [10–12], mainly because they have been replaced by other alternative proposals [13–16]. To advance in the development of alternative methods, collaboration between professional fields is essential. Interprofessional education has been defined by the World Health Organization (WHO) as “*occurring when students from two or more professions learn about, from, and with each other to enable effective collaboration and improve health outcomes*” [17]. It has been identified as a key element in the education of health professions students [18], and incorporated into accreditation and competency standards for several health professions [19, 20]. Pharmacology is a core science course required by multiple health professions [21] in which interdisciplinary collaboration can be integrated, to give students early interprofessional experiences. One of the activities described in this paper is the result of the collaboration between the teachers of the Pharmacology course (Degree in Nursing) and those of Computer Architecture (Degree in Computer Science) with the purpose of increasing students’ motivation and interest in the corresponding subject contents. With this goal in mind, the teachers and students of Computer Architecture programmed the ability of artificial vision and movement in several robots as an alternative method to simulate mice.

In the experience described in the present study, the experimental evaluation of a psychotropic drug, promazine, has served as the basis for analyzing the use of experimental animals in education and research, the ethical and legal aspects of this use, and the importance of interdisciplinary collaboration to achieve alternative methods with Pharmacology students of the Degree in Nursing. In Spain, Pharmacology is a compulsory course of the Degree in Nursing. In this subject, students are provided with knowledge about the actions and properties of drugs so that they can be used with safety and in optimal conditions. Experimental Pharmacology is of great importance within this science, and for this reason we have implemented a practical class focused on the knowledge about the use of experimental animals and the alternative methods employed in pharmacological research. It is necessary for the students to know the importance of the search for alternative methods to replace experimental animals without prejudice to scientific progress [22, 23] and, at the same time, the inability to fully assess some drugs such as psychotropics by using only alternative methods. In their near future, nursing students will become responsible for the handling and administration of medications, as well as for the monitoring of the drug’s effects, and it is important that they have a global vision of all these aspects, including how efficacy and safety of drugs have been characterized previously to be used in humans and how pharmacological data have been obtained from experimental models. Moreover, it is expected that more and more nurses lead or collaborate in research studies in which experimental animals are employed.

Thus, the aim of this study was to assess the perceptions and attitudes of the nursing students enrolled in a Pharmacology course on the use of experimental animals and the possibilities of implementing alternative methods, by comparing the experience carried out for two academic years. For this purpose, we conducted a two-session practical class, in which an interdisciplinary collaboration has also been developed.

## Methods

### Design

A descriptive cross-sectional, quantitative study was conducted. The Strengthening the Reporting of Observational Studies in Epidemiology (STROBE) Statement was used to report data [24].

### Participants

The activity described was intended for students of the Pharmacology course (third semester of the Degree in Nursing, University of Leon). Inclusion criteria for participation in the study were nursing students attending this course in two academic years (2019–20 and 2020–21). No exclusions were applied. There were 104 and 107 students enrolled in the course the academic years 2019–20 and 2020–21, respectively. Of these, 4 and 17 students, respectively, did not follow this practical activity because they had already taken it the previous year. Thus, 190 students (100 in the academic year 2019–20 and 90 in year 2020–21) participated in the study on a voluntary basis.

### Procedure and data collection

**Fig. 1:**
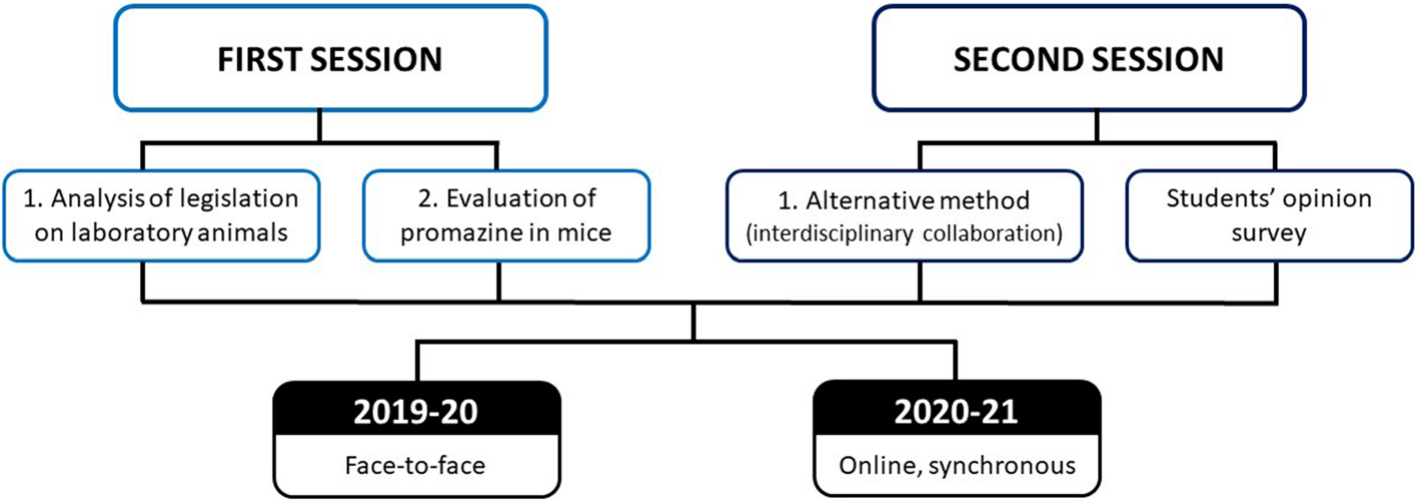
Schedule of the study carried out

A two-session practical class was carried out, focused on the use of experimental animals, the 3R principles and the alternative methods to their use. Figure 1 shows the activities carried out in the two practical sessions and described below. Students were divided into small groups of 10 people to adequately follow up their work. In the academic year 2019–20 all the activities carried out were face-to-face, whereas the academic year 2020–21 synchronous online activities were developed. Contents of the practical class were always the same regardless of it was carried out face-to-face or online.

#### First session: analysis of the legislation for the protection of experimental animals and evaluation of the neuroleptic effect of promazine in mice

The first session started with a teacher’s explanation on the use of experimental animals in preclinical trials and a brief analysis of the European and the Spanish legislation [3, 25]. The goal was to draw student attention to the importance of the use of experimental animals following those regulations established (e.g., no pain caused, reuse of animals in a new procedure, replacement alternatives).

In this session, within the 10-person groups, students were further divided into pairs. Each pair had to analyze and discuss one of the following topics:

- Principles of replacement.
- Reduction and refinement.
- Procedures and the choice of methods.
- Purposes of procedures.
- Classification of procedure severity, and reuse and rehoming.

Once information was collected and analyzed, each pair of students presented their findings to the other students. A discussion was then carried out, acting the teacher as moderator.

After that, the students evaluated the neuroleptic effect of promazine in mice. The objective was not only to learn how to handle mice or to assess the pharmacological effect of this drug, but also to introduce students to the correct use of experimental animals in research studies. For this purpose, students were taught how they should lift, manage and hold mice in a correct and safe way from an animal welfare point of view. Drug administration was made by the teacher and the effect observed by the students.

Male mice of 20–25 g were used. Animals were obtained from the University Animal House. Mice behavior was evaluated by using different tests (chimney, traction, evasion and maze tests) after the administration of saline or promazine. Chimney test was considered positive when the animals climbed backwards up a plastic tube (3 cm inner diameter, 25-cm long) in less than 30 seconds. In the traction test, animals were hung with their forelimbs on a horizontal thin metallic wire suspended ∼30 cm in the air. Those unable to touch the wire with their hindlimbs within 5 seconds were considered positive. In the evasion test, each mouse was kept in a rectangular box with an inclined plane. Two parameters were measured: the time taken to cross a line drawn 5 cm from the flattest area of the box and the number of times the animals crossed this line. As for the maze test, we determined the time taken to cross a line located 5 cm from the entrance of the maze, and the time taken to reach the end of the maze.

As explained before, in the academic year 2019–20 all the activities of this session were face-to-face. To perform the tests and have enough data 6 mice were used in each group and administered saline or promazine. Results were then evaluated as a whole at the end of all groups. In the academic year 2020–21, to promote the replacement of experimental animals and as consequence of the pandemic COVID-19, a synchronous online activity was developed. To replace the face-to-face activity of the previous year, a video, in which a teacher carried out all the tests and explained how to manage and hold the mice, was watched by the students. All students were provided with a table containing the results to calculate and assess the neuroleptic effect of promazine.

#### Second session: presentation of an alternative method with interdisciplinary collaboration and assessment of students’ opinion

To emphasize the need for developing alternative methods to experimental animals, in the second session the students of Pharmacology watched a video produced by the teachers and students from the Computer Architecture course (fourth semester, Degree in Computer Engineering, University of Leon). As explained before, teachers of both courses (Pharmacology and Computer Architecture) worked together to produce this video as a way to improve students’ motivation in their respective subject: programming of artificial vision and movements in robotics to simulate a living being (Computer Architecture students), and assessing the action of drugs in experimental animals and understanding the difficulty of finding alternative methods to evaluate the effects of some drugs (Pharmacology students). The video showed the results of a practical class in which 10 Mindstorms RCX robots were programmed by the Computer Architecture students, under the supervision of their teachers, in an attempt to simulate the usual behavior of a mouse.

**Fig. 2:**
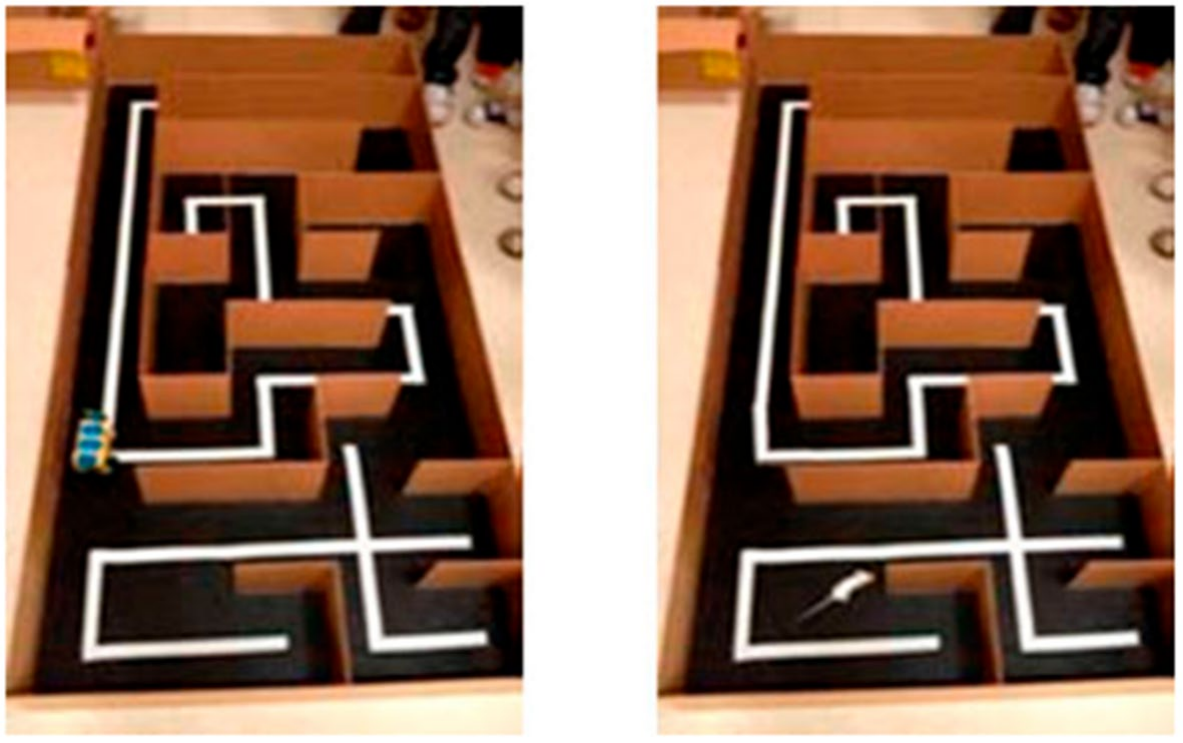
Mouse and programmed robot in the maze

A total of 35 students of this course participated in robot programming, and carried out a pilot assay by applying the maze test to 10 robots and 10 mice. Like the mice, the programmed robots had to go through the maze following a line marked on the floor of the box (artificial vision). They were able to turn (backtracking navigation), if they encountered an obstacle (wall maze), getting robotics-related knowledge (computational vision, artificial intelligence, embedded systems, etc.). The assay was recorded in videos (Fig. 2) provided to the teachers of Pharmacology for later use.

Once watched, a discussion was established among the nursing students on the alternative methods available to replace experimental animals. After that, they answered a questionnaire to assess their opinions. The questionnaire featured 13 questions organized in 2 sections: in the first 1 a 5-point Likert-type scale with 9 questions was used (from 1: strongly disagree to 5: strongly agree). It was previously provided to 10 students who had taken the subject in the preceding academic year (2018–19) and 2 teachers not belonging to the research team to verify that it was well understood. Moreover, Cronbach’s alpha values were calculated for the questionnaires provided in both academic years, and the results were 0.980 in 2019–20 and 0.978 in 2020–21, which shows an acceptable reliability.

In the academic year 2020–21 three new items related to the online development of the practical class and the pandemic scenario were added. In the second part of the questionnaire 4 open-ended questions were used for collecting their opinions and attitudes about the advantages and disadvantages of using live animals (mice) or an alternative method (robots) in the practical class and, by extension, in education. Questions were chosen after a comprehensive review of the existing literature on the assessment of learning in Health Sciences courses, and taking into account the content of regulations evaluated in this practice. As described previously, in the academic year 2019–20 the activity was carried out face-to-face, whereas in year 2020–21 the activity was developed online.

Students had the option to opt-out without penalty or disadvantage, as the questionnaire was anonymous, whether it was given on paper (academic year 2019–20) or online (2020–21). Moreover, the teachers who taught the second practical session did not belong to the research team, to reinforce the voluntary participation of the students in the activity.

### Ethical considerations

The study was conducted according to the guidelines of the Declaration of Helsinki. All the students participated in the study on a voluntary basis, and provided their consent at the beginning of the study. In the academic year 2020–21, informed consent was also obtained online prior to conducting the survey. The study was approved by the Ethics Committee of the University of Leon (ULE0382018). The practical session with laboratory animals was performed in accordance with the guidelines of the European and Spanish legislation [3, 25], and according to this legislation, it did not require separate ethics approval for animal experimentation.

### Data analysis

IBM SPSS 26 software was used for analysis of numerical data. Mean, median and standard deviation were calculated when necessary. The reliability of the questionnaire was evaluated using the Cronbach’s α coefficient, as mentioned above. In quantitative variables, normality was assessed by Kolmogorov-Smirnov test. As data were normal, the non-paired *t* test was carried out. In qualitative variables, the Chi-square test was used. A value of *p* ≤ 0.05 was considered as significant.

Regarding the thematic analysis of the 4 open-ended questions on the advantages and disadvantages of using live animals or alternative methods, two members of the research team worked together to manually group information into themes. The coding was then reviewed by another pair of the research team members. Any discrepancies in the coding were discussed among all the researchers involved in the study until a consensus was reached.

## Results

### First session: analysis of the legislation for the protection of experimental animals and evaluation of the neuroleptic effect of promazine in mice

As described previously, in this first session the legislation for the protection of experimental animals was explained. After the analysis of regulations, a discussion was established with the students. They concluded that it was necessary to develop alternative methods for replacing experimental animals when possible and if not, to make an appropriate use of them. The knowledge acquired, especially the importance of the three Rs (Replacement, Reduction and Refinement), were then applied in those experimental procedures carried out with the mice.

In the practical class with experimental animals, students could clearly see the differences in mice behavior and test times after saline or promazine administration. In the academic year 2019–20, the students watched the live practical session and took part in it with the teacher, whereas in year 2020–21 individual results of the previous year were given to the students after having watched the video produced by their teachers. Thus, in both years the students could analyze the data obtained for the two groups of animals, and calculate the means and standard deviations for each test. The students were able to see that the mice injected with promazine were not able to pass either the chimney or the traction test (only 5 and 2% of the animals did, respectively) compared to those that received saline (96 and 98% passed correctly, Chi-square test, *p* ≤ 0.05). In the evasion test, most of the mice with promazine were not able to cross the line drawn, while mice with saline did it on a mean of 23 ± 6.5 times (vs. 2.0 ± 1.9 times in promazine group, *t* test, p ≤ 0.05). Finally, in the maze test most of the mice with promazine were not able to cross the line or enter the labyrinth, whereas mice with saline reached the end of the maze in only 3.5 ± 1.3 min.

### Second session: presentation of an alternative method with interdisciplinary collaboration and assessment of students’ opinion

Maze test was used in this session to emphasize the difficulty of finding an alternative method (robots) to completely replace mice in the evaluation of promazine effects. Students could watch in the video recorded by the students of the Computer Architecture course that the 10 mice reached the goal in a mean time similar to the obtained by the mice in the previous practical class (3.3 ± 1.1 minutes). Regarding robots, 6 out of the 10 programmed reached the goal, but the mean time needed was higher than for mice (4.1 ± 1.4 minutes). Only one robot reached the goal in less time than mice (35 seconds).

After watching the video, all the students attending the practical class completed voluntarily the questionnaires. Thus, the response rate was 100% both years (100 and 90 students in academic years 2019–20 and 2020–21, respectively). Most of them were women with an age of around 19 years, and had previously studied high school (Table 1).

**Table 1:** Sociodemographic characteristics of the sample

Academic year		
	2019–20	2020–21
Age (years) (mean ± SD)	19.3 ± 1.03	19.5 ± 1.82
Gender		
Women	74 (74%)	74 (82%)
Men	26 (26%)	16 (18%)
Previous studies		
High school	92 (92%)	81 (90%)
Vocational training	8 (8%)	9 (10%)

*SD* standard deviation

The medians and interquartile ranges calculated for each question in the first part of the questionnaire are shown in Table 2. In year 2019–20, the students found the practical class very interesting. Their motivation for learning about the use of experimental animals to assess or develop new drugs had also improved. On the other hand, they were able to better understand the concept of the three Rs, and the importance of the research in alternative techniques for learning Pharmacology. In year 2020–21, scores were slightly lower, although students positively evaluated the practical class and its content as well as in 2019–20. In this latter year, three new items were added (no. 10 to 12), and from the answers provided by the participants it can be inferred that nursing students believed that it was important for them to acquire knowledge on the use of experimental animals in Pharmacology, and that they would not ban their use to assess new drugs. Significant differences were found in scores provided by students from both academic years for questions no. 1, 2, 7 and 8.

**Table 2:** Results obtained in the survey (session 1) employed to assess students’ opinion

Statement	2019–20	2020–21	***p***-value
	Median score (IQR)	Median score (IQR)	
1. I have found the class interesting	5 (1)	4 (1)	0.000 ^a^
2. My motivation for learning about the use of experimental animals in the study and development of drugs has increased	4 (1)	3 (1)	0.013 ^a^
3. It has been a useful tool for learning Pharmacology	4 (1)	4 (1)	0.778
4. It has increased my knowledge about the use of experimental animals in the study and development of drugs	4 (1)	4 (1)	0.909
5. It has helped me to know how to value the use of experimental animals in Pharmacology	4 (1)	4 (1)	0.952
6. It has been useful for me to know the legislation on experimental animals	4 (1)	4 (1)	0.077
7. It has helped me to understand the concept and the importance of the practical application of the three Rs (Replacement, Reduction and Refinement)	4 (1)	4 (1)	0.023 ^a^
8. I consider important the research in alternative techniques for learning Pharmacology	5 (1)	4 (1)	0.021 ^a^
9. I consider interesting the interdisciplinary collaboration in the teaching of Pharmacology	4 (1)	4 (1)	0.782
10. I would have liked to be able to do the practice with mice, instead of watching a video	–	5 (1)	
11. As a nursing student I do not need to know the use of experimental animals in Pharmacology	–	2 (1)	
12. If I could legislate, I would ban the use of experimental animals in the research of new drugs	–	2 (1)	

Median score of a range from 1 (strongly disagree) to 5 (strongly agree). IQR: Interquartile range

^a^ Significant differences between both academic years (Chi-square test; *p* ≤ 0.05)

The four open-ended questions in part 2 allowed the students to give their opinion about the advantages and disadvantages of using experimental animals (mice) or an alternative method (robots). Table 3 includes those comments made by the students in the second part of the survey. Opinions were coded by the teachers taking into account the most frequently expressed ideas in the answers.

**Table 3:** Opinions given by the students on advantages/disadvantages of mice/robots use (academic years 2019–20/2020–21)

	Mice	Robots
Advantages	Knowledge of reality (78%/80%) Reliability (67%/69%) Similar to humans (51%/56%)	Avoids the use of animals (65%/73%) Animals do not suffer (61%/58%) Scientific progress (48%/60%)
Disadvantages	Potential damage to the mouse (92%/89%) Wide variations in responses (71%/69%)	You do not see the real effect (91%/93%) High cost (72%/63%) Difficult to program (54%/68%)

The answers given showed that students believed that the knowledge of reality was the main advantage of the use of mice. As for robots, they avoided the use of animals. On the other hand, the main disadvantage for the use of mice was the potential damage that they could potentially cause by handling or injecting saline/promazine (even if they had carried out the procedure correctly), and for robots, that it was more difficult to evaluate the real effect of a drug.

## Discussion

A pedagogically effective approach should involve a variety of teaching strategies. The results of our study indicate that different learning strategies can be integrated in a coherent way, and that this implementation had a clear positive impact on the engagement of our nursing students with the scientific and ethical aspects on the use of animal experimentation.

The use of experimental animals for scientific or educational purposes always generates an intense debate [26]. Main concerns about their use are related to their welfare (causing them pain, distress, suffering, lasting harm or even death) and to the failure of animal models to adequately represent human disease. Most scientists agree that animal research should be permitted as long as it is carried out for good reason, using human conditions as much as possible, if there are no viable alternatives and under strict regulation [1, 2, 27].

As explained before, procedures used to evaluate promazine effects are considered as not harmful according to Directive 2010/63/EU [3], and they would be a good basis to explain the regulations on animal protection, highlighting the aspects related to animal replacement. Various alternative methods to the use of experimental animals in research and education have been suggested and accepted worldwide [28–30].

Measurement of students’ opinion is a way to evaluate educational programs [31–33]. As can be seen in Table 2, the scores of the different questions in part 1 were near 4 points, indicating that our students rated the activity positively. The median scores of 5 for items 1 (“I found the class interesting”) and 8 (“I consider important the use of alternative techniques for learning Pharmacology”), suggest that the practical class achieved its goals in the academic year 2019–20. In year 2020–21, the highest score was obtained in item 10 (“I would have liked to be able to do the practice with mice, instead of watching on a video”, 5 points), which may be influenced by the health emergency lived by the students, including the perception of the urgent need to develop a new drug or a vaccine against COVID-19 [34–36]. These results indicate that students prefer to do the practice themselves instead of watching a video, and that they are interested in this topic, not rejecting the use of experimental animals. Other studies have revealed that male students seemed to be more positive toward animal use in research [37, 38]. However, we have not been able to compare students’ attitudes by gender due to the low number of male students enrolled in this Degree.

In a survey carried out among students of different degrees, most of them found animal research as morally acceptable, with a 78% of acceptance among nursing undergraduates. Moreover, they believed that this type of research played a significant role in treating human diseases [38]. Nevertheless, in the study of Elhaji and Basheti [33] 49.5% students refused to handle animals, being phobia the main reason to explain this rejection.

The opinions provided by the students in the second part of the survey (Table 3) revealed that, similarly to other authors [10, 27, 39], students considered “causing them pain” as the main concern for the use of live animals, even though they were assured that the procedure was correctly carried out. Similar concerns were reported by other authors [40, 41]. Our students believed that the use of alternative methods (robots) would avoid animal suffering. Thus, the practical experience implemented has been successful in making the students see the intrinsic value of experimental animals, and that they should be always treated as sentient creatures, restricting procedures in which they are used to those that ultimately benefit human or animal health [3].

The humane use of experimental animals is of vital importance to understand the mechanisms of health and disease, as well as to develop therapies and promote health. As other authors, we believe that bioscientists’ formation should include, in addition to scientific knowledge, a thorough evaluation of the moral implications of scientific research [42]. An increasing number of nurse researchers use animals to find solutions that can be later applied to patients. The experience described may be also seen as a first training step to provide nurses with the skills necessary to become nurse bioscientists, giving them a more visible role in basic and traslational research. However, although there is much work to be done to translate the animal research to the nursing practice, it is important for nursing students to know the research with animals used to develop new drugs and vaccines that they will be responsible for administering.

Regarding the use of the robots as an alternative method, simulation resources have been used in Pharmacology teaching for several years to help students understand difficult contents, and to demonstrate its application in clinical situations [21, 43]. The purpose of using robots was to highlight the importance not only of the replacement of experimental animals but also of interdisciplinarity. The development of these methods needs an interdisciplinar collaboration and many times computers are used to help in this task.

In this sense, interprofessional education has been identified as an essential element in the education of health professions students [21, 44]. This is an ongoing process, and it is necessary to learn how to work side-by-side, dealing with different aspects of a single problem, with the insights and methodologies from a variety of disciplines, and a broader perspective in relation to health practice. Students should be taught that no single discipline can claim the ownership of a topic. Recently, the American Association of Colleges of Nursing Position Statement on interdisciplinary education and practice aims to educate nursing students in such a way that promotes joint interdisciplinary planning, decision-making, and goal setting [45].

The interdisciplinary collaboration carried out has served to convey to the students how hard it is to design an alternative method to an experimental animal (robot) in which the actions of a psychic depressor on its motor activity can be assayed. Nursing students not only appreciated the difficulty of simulating the behavior of a living being, but also the importance of the interdisciplinary collaboration and the limitations and difficulties of developing these methods [26, 46–48]. Together with their teachers, students came to the conclusion that, in the future, computer professionals could implement software to simulate different situations of central nervous system affectation caused by stimulant or depressant drugs with the advice of Health Sciences professionals like them.

Regarding the robots employed in the activity, the teachers of the Architecture Computers course chose the Mindstorms RCX ones as they accept a good variety of programming languages and they were especially designed for educational purposes [49–51]. As recommended by the teachers of the Computers Architecture course, we explained that robots did not know the right way, and they should explore like mice: choosing a path to get to an intersection and turning back to test another option if the path chosen is wrong. As it occurs at each intersection, the complexity of the algorithm grows rapidly [50]. The implemented activity served our students to verify that at the present time, alternative methods cannot completely replace experimental animals in the evaluation of new drugs, and that it is necessary to use them in some phases, always ensuring a high level of protection for animals [22, 23]. In a future, properly programmed robots or other in silico options could be used in the experimental assessment of a drug acting on the central nervous system. Our students found that it would be possible to simulate the behavior of a mouse under the effects of psychotropics but with intense programming work [52].

Although the survey offers insight on the perceptions of students about the use of experimental animals and alternative methods, it is not exempt from limitations. In this sense, the study was conducted in only one course (Pharmacology) and using in both academic years one cohort of undergraduate students. Moreover, students may have been, in some way, directed toward one type of response or another taking into account the information provided from the teachers.

Further studies are needed to evaluate the knowledge that nursing students have on this topic when they are in higher courses, once they have taken other subjects and developed part of their practical activity in hospitals and health centers. On the other hand, the improvement of the robot described, or other in silico possibilities, would make possible to simulate the action of other drugs, which in turn would help to reinforce the interdisciplinary activity of Informatics and Nursing.

## Conclusions

The alternative method to experimental animals designed for the teaching of Pharmacology practices, with the collaboration of the teachers and students of a different discipline (Computers Architecture), was useful for the students to understand and better accept the employment of experimental animals in biomedical research and education, as well as to know the legislation applied in the protection of animals. The development of this teaching experience demonstrates the importance of interdisciplinary collaboration in education, and the need of strengthening it from the beginning of nursing education. Moreover, as part of this educational experience has been carried out online under pandemic, we have seen that students prefer face-to-face practical activities and to do the practice themselves.

The results of our study reveal that it is possible to design and carry out interdisciplinary and active strategies to draw students’ attention to a certain topic. The challenge of developing a new and original teaching tool is an unusual situation for students, which leads them to find answers that are not in the textbooks, improving their creativity and interest in the topic developed in the class. We believe that the use of experimental animals and alternative methods has been successful in achieving the goals initially set. This teaching proposal can be applied in any classroom, of any country, with the only change of the corresponding legislation, to reinforce students’ skills and confidence with regard to animal experimentation.

## Data Availability

The datasets used and/or analysed during the current study are available from the corresponding author on reasonable request.

## Abbreviation

STROBE: Strengthening the Reporting of Observational Studies in Epidemiology
